# A Sketch of Language History in the Korean Peninsula

**DOI:** 10.1371/journal.pone.0128448

**Published:** 2015-05-29

**Authors:** Sean Lee

**Affiliations:** Department of Biological Sciences, School of Science, The University of Tokyo, Tokyo, Japan; St. Petersburg Pasteur Institute, RUSSIAN FEDERATION

## Abstract

Among 7100 languages spoken on Earth, the Koreanic language is the 13th largest, with about 77 million speakers in and around the Korean Peninsula. In comparison to other languages of similar size, however, surprisingly little is known about the evolution of the Koreanic language. This is mainly due to two reasons. The first reason is that the genealogical relationship of the Koreanic to other neighboring languages remains uncertain, and thus inference from the linguistic comparative method provides only provisional evidence. The second reason is that, as the ancestral Koreanic speakers lacked their own writing system until around 500 years ago, there are scant historical materials to peer into the past, except for those preserved in Sinitic characters that we have no straightforward way of interpreting. Here I attempt to overcome these disadvantages and shed some light on the linguistic history of the Korean Peninsula, by analyzing the internal variation of the Koreanic language with methods adopted from evolutionary biology. The preliminary results presented here suggest that the evolutionary history of the Koreanic language is characterized by a weak hierarchical structure, and intensive gene/culture flows within the Korean Peninsula seem to have promoted linguistic homogeneity among the Koreanic variants. Despite the gene/culture flows, however, there are still three detectable linguistic barriers in the Korean Peninsula that appear to have been shaped by geographical features such as mountains, elevated areas, and ocean. I discuss these findings in an inclusive manner to lay the groundwork for future studies.

## Introduction

A penetrating insight that evolution can describe historical change of languages has enabled us to chart out how language history unfolds within the episodes of human history, as explosive as the Austronesian languages of the Pacific [1,2] or as complex as the Indo-European language family of the European continent [3,4]. Considering, however, that we have managed to quantify historical changes of only a dozen or so language families [5,6] out of more than 260 families around the world [7], it is obvious that the study of language evolution must continue its efforts to excavate and quantify as many world language histories as possible. The questions of “how” and “why” something evolves, after all, can be fully answered only when we have an exhaustive description of “what” it is [8].

In this paper, I introduce a new set of lexical data sampled across the Korean Peninsula and quantitatively describe its evolutionary characteristics in an attempt to further our understanding about the linguistic history of the Koreanic language. Unlike other languages of similar size and presence, almost nothing is certain about the evolutionary history of the Koreanic language. This is because much of what we know about the language comes from materials written after the invention of the Korean alphabet, namely Hangul, in 1446. This means that the language's change and variation prior to Hangul can only be inferred indirectly from historical records, toponyms, and popular literature [9,10]. This indirect inference is further complicated by the fact that much of the information is preserved in cryptic characters of Sinitic, a prestigious written language adopted across Asia much like Latin in Europe. In addition, the genetic relationship of the Koreanic to other neighboring languages is still unresolved and widely speculated, from being a branch of large families such as Altaic [11] or Koreo-Japonica [12] to being a language isolate with no demonstrable relatives [13]. This uncertainty prevents us from triangulating the unknown points in the Koreanic language history through contrasting its characteristics with those of sister languages. This therefore nullifies the comparative method, which is the gold standard strategy for untangling the past of languages. Due to these disadvantages, the Koreanic language has rarely been a popular subject of systematic empirical study, and the results presented in this paper are thus an attempt to lay the first stone.

**Fig 1:**
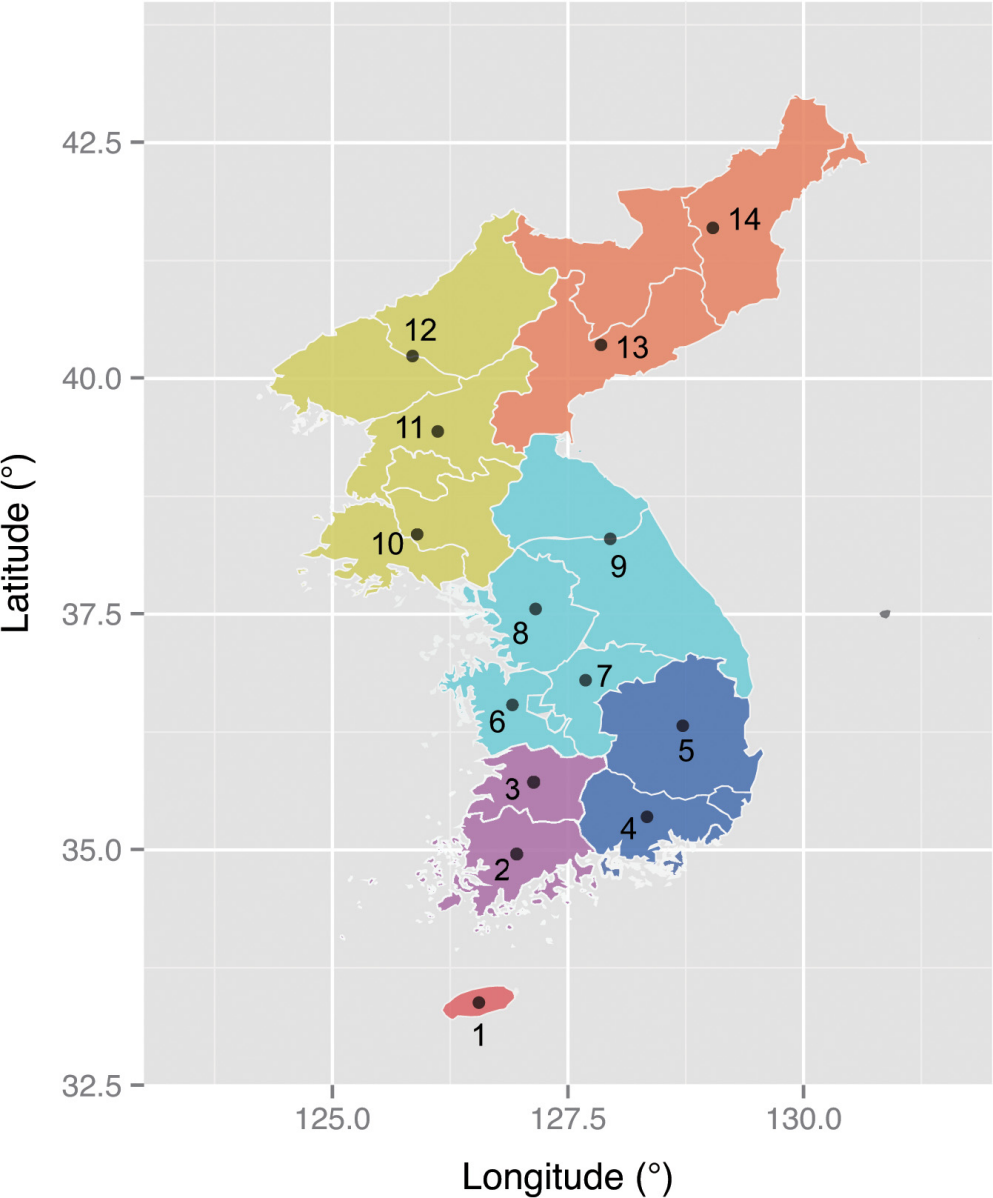
Map of the Koreanic language variants. Colored areas represent the conventional subgrouping scheme for the Koreanic language variants (Orange-Hamgyong, Yellow-Pyongan, Aqua-Central, Blue-Gyeongsang, Purple-Jeolla, Red-Jeju). Sample locations: 1-Jeju, 2-Southern Jeolla, 3-Northern Jeolla, 4-Southern Gyeongsang, 5-Northern Gyeongsang, 6-Southern Chungcheong, 7-Northern Chungcheong, 8-Gyeonggi, 9-Gangwong, 10-Hwanghae, 11-Southern Pyongan, 12-Northern Pyongan, 13-Southern Hamgyong, 14-Northern Hamgyong.

The current study compiles a total of 2316 lexicons from 15 Koreanic language variants (Fig 1) and describes their historical characteristics using statistical methods adopted from evolutionary biology. Leveraging the historical linguistic data with quantitative tools, the current study demonstrates a non-treelike evolution of the Koreanic language variants as well as a high degree of linguistic homogeneity within the Korean Peninsula. These features seem unusual for a language that has a large speech community with several thousand years of population history [9,14]. Triangulation with recent evidence from genetic [15] and human mobility [16] studies, it is argued here that these observations might be reflecting complex and continuous human migrations within the Korean Peninsula. Despite the complex history, the data reveals signatures of several major linguistic barriers in the Korean Peninsula. These barriers appear consistent with the recent findings that linguistic diversity, like biological diversity, is partly shaped by the physical environment [17,18].

## Materials and Methods

Two hundred and forty six (246) basic vocabulary items [19,20] were extracted from each of 14 living and one (1) ancient Koreanic variants using multiple sources: (i) a large field collection made by Shimpei Ogura [21], (ii) a modern dictionary of Koreanic variants that combine lexicons from several different references [22], and (iii) an etymological glossary of Middle Korean that contains lexicons sampled from over 240 historical documents [23]. Every attempt was made to identify the cognate candidates [24] through checking against the known cognates and sound correspondences [9,10,25–27]. An ideal cognate dataset, however, requires several rounds of independent verification to ensure that all homologous words are thoroughly identified; and because the data compiled here is the first of its kind, it is possible that the data may potentially carry a small amount of undetected error. I therefore make the data openly accessible along with the findings (S1 Dataset), so that the community can examine and modify the cognate candidates if necessary.

BEAST 1.8.2 [28] was used to carry out Bayesian phylogenetic analyses on the cognate sets. Multistate meaning-based cognate sets were transformed into binary states indicating presence (‘1’) or absence (‘0’) of a cognate for each lexicon. The result was a 15 by 383 matrix (S1 Dataset). The evolutionary rate and branch lengths were calibrated with two priors. The first calibration consisted of a tip-date sampling prior assigned to Middle Korean with a normal distribution (mean: 500, standard deviation: 100). This prior was based on the information that Middle Korean was spoken around the 15th century [9]. The prior distribution was truncated according to the oldest and the earliest sampling ages (870 and 87 years ago respectively) of the original documents [23]. The second calibration was assigned to the clock rate parameter with a lognormal distribution prior (mean: 3e-4, standard deviation: 0.7). This prior was based on the clock rates empirically estimated from Japonic [29] and Ainu [30] languages under various model settings. A series of analyses was carried out to compare the fit [31] between a simple Markov chain substitution model and covarion model [32] with either a strict clock or a relaxed clock [33]. A correction for ascertainment bias was applied. Stochastic Dollo model [34] was not used here because it was reasoned that using substitution models with reversible transition between absence or presence of a cognate would be a more conservative approach than allowing only a single transition from absence to presence under the stochastic Dollo model. In addition, the NeighborNet algorithm [35] and Hamming distance were used for an explicit visualization of phylogenetic signal. Reticulations in the resulting network indicate the amount of conflicting phylogenetic signal among language samples.

The internal structure of the Koreanic variants as well as the variants with admixed ancestry were inferred by using a Bayesian clustering model implemented in STRUCTURE 2.3.4 [36]. In the context of language evolution, admixture can be conceptualized as language variants being born from two or more ancestors due to hybridization between distinctive speech communities [4,37,38]. A common form of this phenomenon might be observed from languages spoken by adult second-language learners who strive to assimilate into a different ethnolinguistic group. An extreme form of the linguistic admixture might be nativized pidgin, or creole language. The clustering model in STRUCTURE assumes that individuals (language variants) represent a mixture of *K* populations in which each population is characterized by a set of allele frequencies (cognates) at each locus (basic vocabulary item). The algorithm then iteratively assigns the individuals and their allele frequencies into *K* clusters, so as to minimize departure from both Hardy-Weinberg equilibrium within populations and linkage equilibrium between loci. The algorithm estimates the amount of admixture among populations by allowing some proportion of each individual's genotype (lexical makeup) to be assigned to more than one population. Following the previous research [39,40], the data was processed as haploids without inferring admixture linkage disequilibrium. From 100 independent runs (10,000 burn-in and 20,000 iterations) for each of 1 to 15 *K* populations, the best-fitting number of *K* was selected with the mean likelihood and Delta K [41,42].

Finally, Barrier 2.2 [43] was used to explore how geographical features have shaped the lexical diversity in the Korean Peninsula. Given that the language samples are located on a two-dimensional surface, the Delaunay triangulation method [44] can derive a geometric network that connects the samples with a set of triangles and closed Voronoi diagrams [45]. Given such a network, each edge can be assigned a pairwise distance measure (Jaccard was used here) between samples. Following the assignment of distance measures, Monmonier’s maximum difference algorithm [46] draws a barrier by locating an edge with the maximum value and proceeding to an adjacent edge with the next maximum value, until it reaches the limit of the network or forms a loop. Five barriers were estimated initially (i.e., the minimum number of barriers possible under the conventional subgrouping scheme [27]), and Mantel r^2^ values estimated from an isolation-by-barrier approach [17] (i.e., applying indicator variables to separate samples connected by geography from those disconnected by physical barriers) were used to determine the appropriate number of barriers as well as whether or not they remain statistically significant after controlling for geographical proximity [47–49].

## Results and Discussion

**Fig 2:**
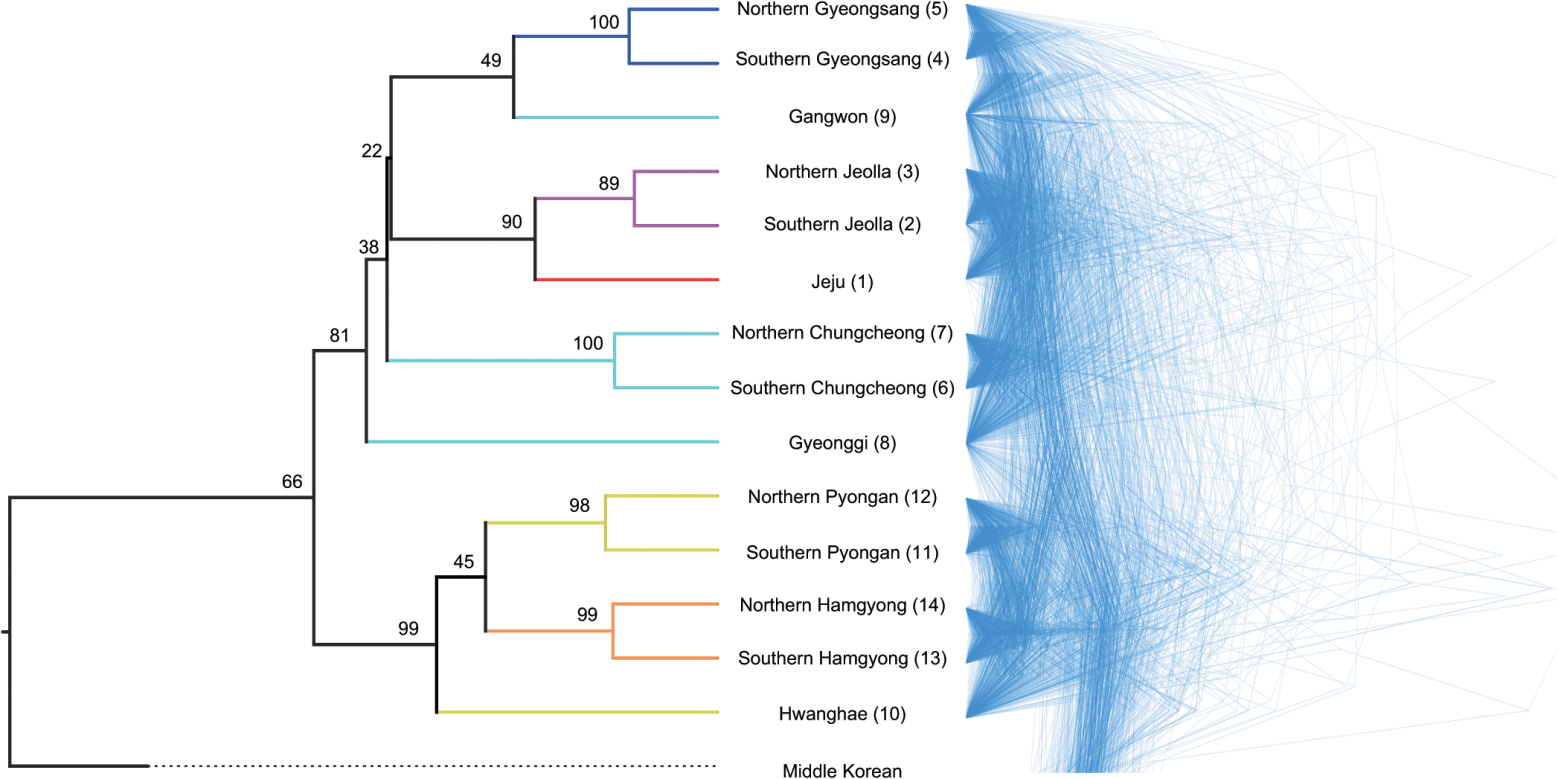
Phylogenies of 15 Korean language variants. (A) Left panel shows a maximum clade credibility tree. Branches are colored according to the conventional subgrouping scheme (Orange-Hamgyong, Yellow-Pyongan, Aqua-Central, Blue-Gyeongsang, Purple-Jeolla, Red-Jeju). All node heights are scaled to match the posterior median node heights. The value on each branch is the posterior probability, showing the percentage support for the following node. (B) Right panel shows a cloudogram of consensus trees. Branch lengths of each consensus tree represent the average branch lengths of all sampled trees with the same topology. The low node supports in the maximum clade credibility tree and the lack of consistency in the cloudogram indicate that the evolution of the Koreanic language variants is far from treelike. Estimated divergence times are not shown because they may potentially be misleading. Numbers in the tip labels match the sample locations in Fig 1.

Bayes factor tests strongly supported the covarion substitution model with a relaxed clock as the best fit, with Bayes factors of 24 over the second best model using the covarion with a strict clock (S1 Table). Fig 2 shows the results of the best-fitting model. When we inspect two different ways of representing the resulting trees as shown in Fig 2, one characteristic stands out immediately: the lack of stable hierarchical structure among the 15 Koreanic variants. The left panel of Fig 2 shows that the estimated node supports are robust only for the first order ancestors of the modern variants, and the certainty of phylogenetic relationships decreases with the nodes (i.e., estimated most recent common ancestors) getting closer to the root. Also, the expected clades according to the conventional subgrouping scheme [27] were largely unrecoverable. The lack of hierarchical structure and the high degree of phylogenetic uncertainty manifest even more clearly when consensus trees are visualized as a cloudogram [50] as shown in the right panel of Fig 2. The non-existence of agreement on branching patterns among the consensus trees indicate that the evolution of the Koreanic language variants is far from treelike (divergence times are not shown as they may potentially be misleading, but all estimates can be reconstructed with S2 Dataset).

**Fig 3:**
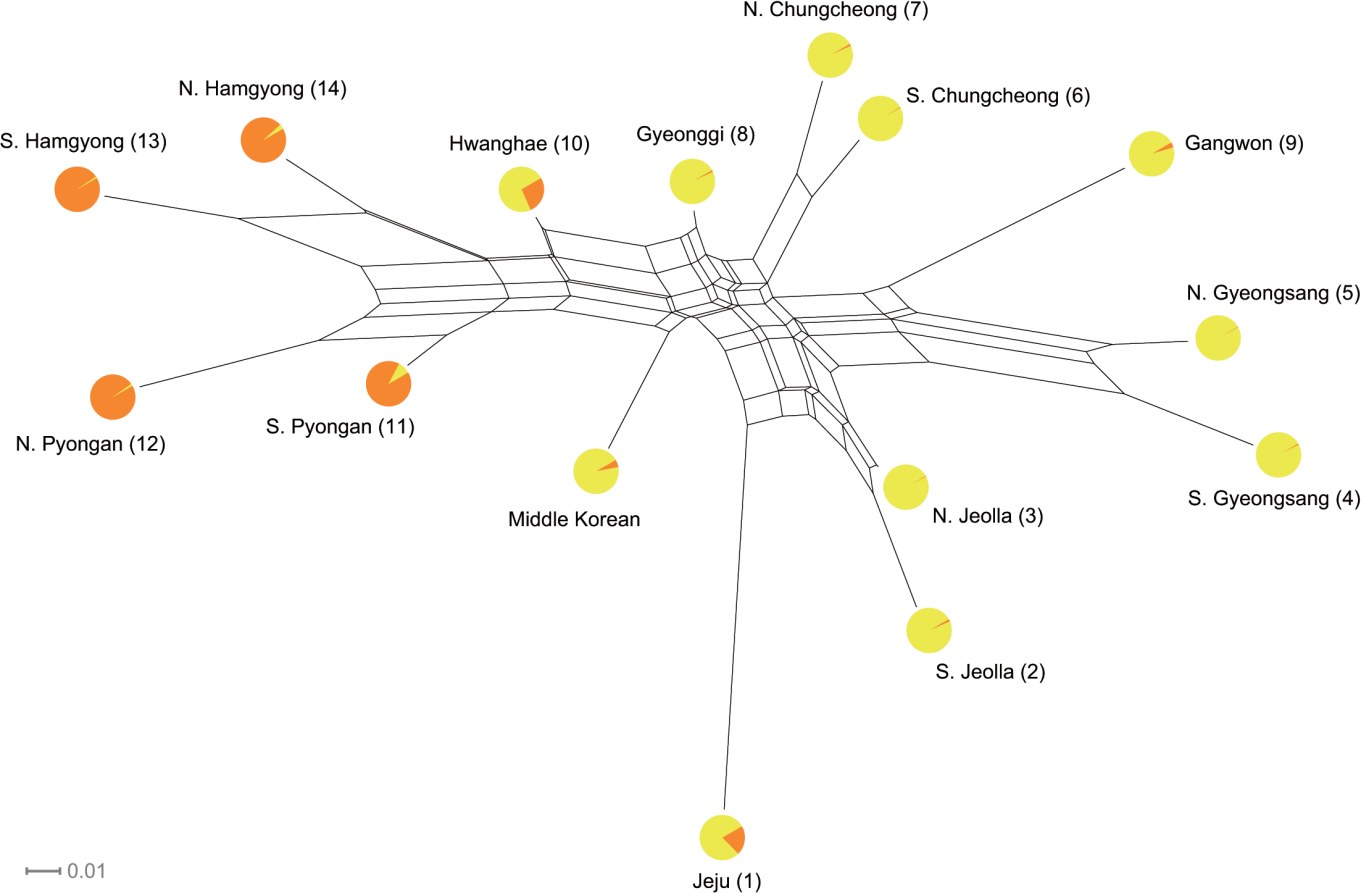
Split graph showing the results of NeighborNet and STRUCTURE. Reticulations indicate presence of conflicting signal, which can be interpreted as admixture, horizontal transmission, and rapid radiation. Circles at the tips represent the amount of admixture between clusters (*K* = 2) estimated by STRUCTURE. Numbers in the tip labels match the sample locations in Fig 1.

Subsequently, all relationships among the Koreanic variants were visualized in a split graph. The reticulations in Fig 3 clearly show conflicting phylogenetic signal as expected by the lack of hierarchical structure in the Koreanic language tree. It is, however, difficult to ascribe the observed reticulations to a specific cause because they may potentially stem from a combination of three factors: hybridization between distinctive variants giving rise to mixed variants (i.e., admixture [37]), movement of linguistic features between variants by non-vertical transmission (i.e., horizontal transmission [51]), and abrupt breaking up of an ancestral language into several offspring (i.e., rapid radiation [4,52]). In order to understand the historical factors responsible for the observed reticulations, two types of inference were made. First, a Bayesian cluster analysis was used to estimate the precise extent to which the reticulations are stemming from admixture events among the Koreanic subgroups. The estimated mean likelihood and Delta K (S2 Table) both indicated that, in contrast to the conventional scheme of six subgroups [27], there are only two unique language clusters in the Korean Peninsula with a small amount of admixture between them (i.e., only Hwanghae and Jeju having more than 10% of admixture). These results are shown as pie graphs overlaid on the split graph (Fig 3). If the split graph and the results from cluster analysis are interpreted in an inclusive manner, then it seems highly unlikely that sporadic admixture events among six distinctive subgroups is giving rise to the observed reticulations; rather, it seems more likely that the observed reticulations are reflecting inconsistent evolutionary signals within the two linguistically homogenous clusters, possibly caused by prevalent horizontal transmission and/or rapid radiation.

As there is yet no statistical method to distinguish between the reticulations caused by horizontal transmission from those caused by rapid radiation, the second type of inference was made through triangulating the current results with other lines of evidence. Analyses of mitochondrial DNA [53], Y-chromosome STR [54], and genome-wide SNP [15] all indicate that there is no statistically significant population structure within the Korean population (as estimated by F_ST_ or STRUCTURE) except for a small difference between Jeju Island (sample location 1) and the rest of the region [53,54]. This consistency between the genetic and the current findings allows us to infer that the observed linguistic/genetic homogeneity might be pointing to a common mechanism underlying both domains, and thus a particular historical process that explains one domain might also explain the other as well. If we subsequently include a third line of evidence to this framework, then it becomes apparent that the strongest candidate for the hypothesized common mechanism is a complex and continuous horizontal gene/culture transmission within the Korean Peninsula. The Koreanic speech community has a long tradition of keeping genealogical records, called Jokbo, that exhaustively keep track of all individuals of a family clan including who they are married to (in terms of patrilocality). A recent study [16] taking advantage of this genealogical information revealed that, for at least last 750 years, women seemed to have married almost any men in the Korean Peninsula, meaning that the migration of Koreanic speaking females was geographically unbounded, resembling a random-mating population in evolutionary biology. Moreover, it was also suggested that if the discrepancy between the ancestral and current geographic distributions of the patrilocal clans is to be explained by a simple diffusion process from the clan homelands to the current residents, then it is estimated to have taken around 67,000 years for the clans to be as geographically mixed as they currently are. This means that the population history of Koreanic speaking males is characterized by frequent relocation of residents (i.e., abandoning homelands). If we are correct to think that these three lines of evidence point to the same historical process, then a high degree of reticulations and homogeneity among the Koreanic variants could be due to migration-triggered horizontal gene/culture transmission. In other words, the geographically unrestricted gene/culture flows might have wiped out any accumulated traits that differentiate subgroups, and contributed to the observed homogeneity and the uncertainty of the tree representation.

It is difficult at this stage to pinpoint exactly what is responsible for the hypothesized intensive migrations. Interestingly, however, Sinitic languages have previously been argued as a prime example of the internal human migrations obscuring the hierarchical relationships [55], and it has been thought that the major factors that triggered those migrations were war, natural disaster, famine, and change in economic landscape [56]. Considering that the Sinitic and Koreanic languages are the closest geographical neighbors, it can be hypothesized that their history may be intertwined by similar evolutionary forces, such as decline in food production due to climate change in the region, causing a major population drop and rapid opening up of new niches for the surviving populations to move into. On a broader level, it has previously been argued that language evolution on a global scale go through a cycle of two modes of change: a punctuation mode in which languages diverge from one another in a clear treelike fashion, and an equilibrium mode in which languages of an area blur their own hierarchical structure by exchanging linguistic features among themselves [57]. Along this line of thought, I speculate that the Koreanic and Sinitic languages could be taken as examples of the equilibrium mode of language evolution. Accumulation of more quantitative descriptions of similar cases from around the world would allow us to identify the shared factors that explain how and why this phenomenon occurs.

**Fig 4:**
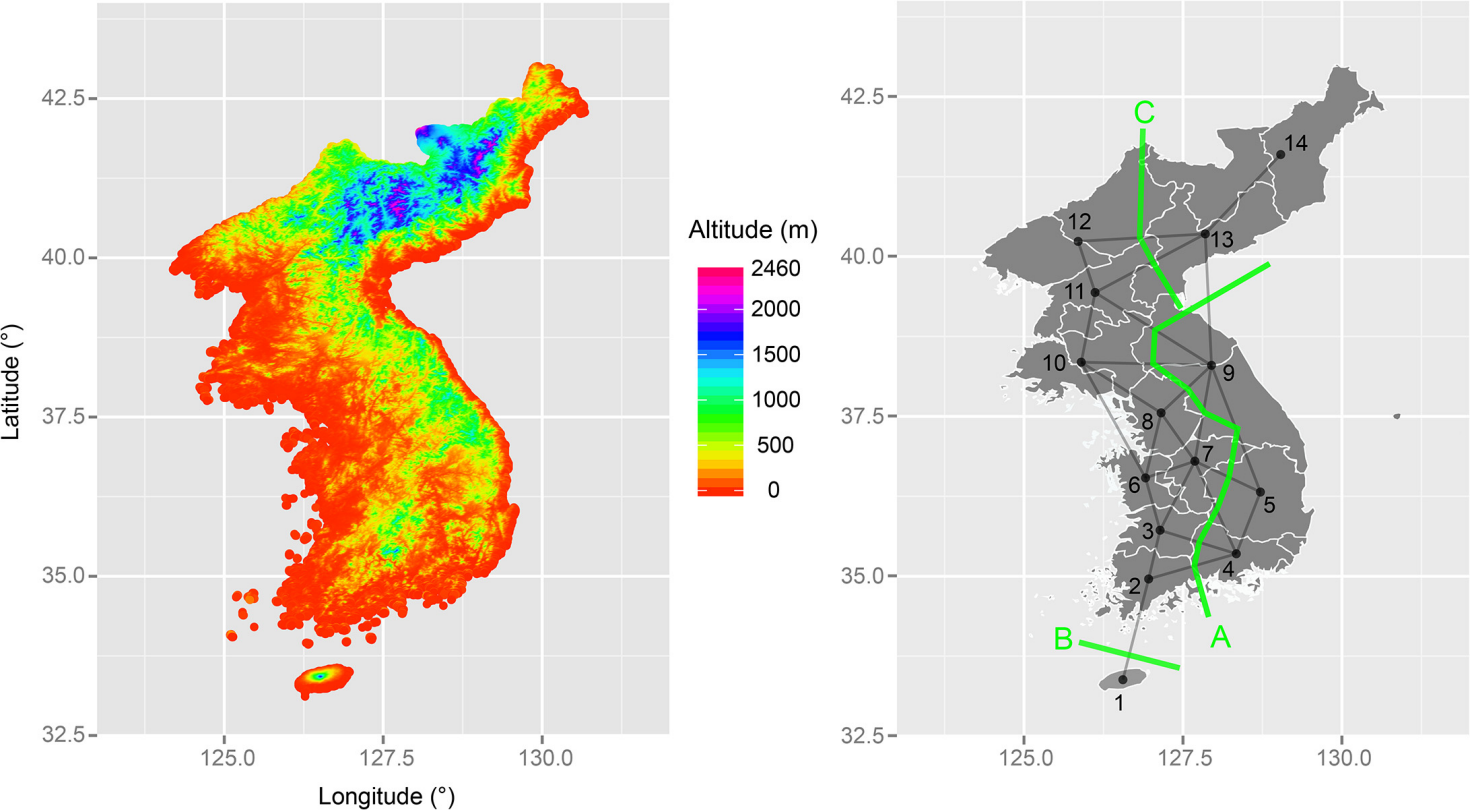
Maps showing the geographical features and linguistic barriers. (A) Left panel shows point-by-point estimates of altitude in the Korean Peninsula. (B) Right panel shows the result of Barrier analysis on a geometric network of sample locations. Sample locations: 1-Jeju, 2-Southern Jeolla, 3-Northern Jeolla, 4-Southern Gyeongsang, 5-Northern Gyeongsang, 6-Southern Chungcheong, 7-Northern Chungcheong, 8-Gyeonggi, 9-Gangwong, 10-Hwanghae, 11-Southern Pyongan, 12-Northern Pyongan, 13-Southern Hamgyong, 14-Northern Hamgyong.

Despite the complex history of horizontal transmission in the Korean Peninsula, a simple computational geometry approach was able to detect linguistic barriers that show close correspondence with geographical features such as mountains, elevated regions, and ocean (Fig 4). Five barriers were estimated initially (S1 Fig), and then increase in Mantel r^2^ was observed with each barrier addition to explain the difference in lexical beta diversity [58] between the variants connected by geography and those separated by a barrier. When the estimated r^2^ values were plotted and interpreted in a manner similar to a scree plot (S2 Fig), it became apparent that addition of more than the first three barriers led to only a negligible increase in r^2^ for Pearson correlation and no increase for Kendal rank correlation. It was thus reasoned that only the first three barriers are meaningful. All r^2^ estimates remained statistically significant after controlling for geographical proximity (S3 Table). The left panel of Fig 4 maps point-by-point estimates of altitude of the Korean Peninsula on a longitude-latitude grid, and the right panel shows the estimated barriers on a geometric network of the Koreanic variants. By overlapping these two maps, it can be observed that (i) barrier A matches the Sobaek Mountains that run between Gyeongsang variants (sample locations 4 and 5) and Jeolla variants (locations 2 and 3) as well as the Taebaek Mountains that put Gangwon variant (location 9) in an elevated area, (ii) barrier B runs along the Jeju Strait that physically isolates Jeju variant (location 1) from the rest, and (iii) barrier C corresponds to the Rangrim (Nangnim) Mountains that draw a border around the highlands occupied by northern and southern Hamgyong variants (locations 13 and 14). If these interpretations are correct, then they are in agreement with the recent findings that the way in which languages diversify over time is deeply intertwined with the landscape that they occupy [17,18]. Curiously, the identified barriers show no correspondence to the estimated language clusters (Fig 3). I speculate that the reason for this difference is that the degree of beta diversity defining the linguistic barriers is small, and only an algorithm that focuses on the maximum difference, however small, can pick up the signal in the data. If the estimated barriers are correct, then it can be suggested that there are two barriers in the yellow cluster (i.e., southern and central variants) and one barrier in the orange cluster (i.e., northern variants) shown in Fig 3. Although the patterns between the two results cannot be fully reconciled at this point, I argue that the discrepancy actually gives us some confidence that one of them is unlikely to be an epiphenomenon of the other.

## Summary and Conclusion

The results presented here indicate that the early historical relationships among Koreanic variants are considerably non-treelike. This makes it unlikely that other tree-based inferences (e.g., phylogeographic analysis) would make any further contributions. Although all other possibilities cannot be ruled out completely, it appears that the strongest candidate for the cause of a weak higher-order structure as well as a high degree of homogeneity within the Korean variants is intensive gene/language flows triggered by geographically unrestricted migrations. Despite the dynamic demic history, major geographical barriers in the Korean Peninsula seem to have left detectable signatures in the pattern of lexical diversification, suggesting a strong tie between the evolution of Koreanic variants and the geographical features of the Koreanic Peninsula.

Similar to many other ethnolinguistic groups in Asia [59], the rise of national identity as well as rapid change in the economic landscape have led the Koreanic speakers to set on an evolutionary course that can seriously threaten its internal linguistic diversity. Therefore, our chance to understand the Koreanic language history is diminishing fast. While it is fully recognized that the results presented here cannot readily contribute to answering more fundamental questions such as the origin and expansion of the Koreanic language, it is hoped that the efforts made here to bring the Koreanic language history into the realm of language evolution would provide a good starting point for future investigations. Accumulation of more historical linguistic data, both modern and ancient, will allow us to better understand the dynamics of language evolution in the Korean Peninsula, and its place within a larger scheme of language evolution in Asia.

## Supporting Information

S1 Dataset All data used in the study. (XLSX)

S2 Dataset BEAST and NEXUS files. (ZIP)

S1 Fig The raw output from Barrier analysis. (EPS)

S2 Fig Relationship between the number of barriers and the amount of lexical beta diversity explained. (EPS)

S3 Fig Bar plots showing the amount of admixture for *K* = 2–5. (EPS)

S1 Table Marginal likelihoods estimated from all models. (XLSX)

S2 Table Mean LnP(K) and Delta K for each of *K* = 1–15. (XLSX)

S3 Table All results from Mantel tests before and after controlling for geographical proximity. (XLS)

## References

[1] Gray RD, Drummond AJ, Greenhill SJ. Language phylogenies reveal expansion pulses and pauses in Pacific settlement. Science. 2009;323: 479–483. 10.1126/science.1166858 19164742

[2] Blust R. The Austronesian languages. Canberra: Asia-Pacific Linguistics; 2013.

[3] Gray RD, Atkinson QD. Language-tree divergence times support the Anatolian theory of Indo-European origin. Nature. 2003. 10.1038/nature02029 14647380

[4] Ringe D, Warnow T, Taylor A. Indo‐European and computational cladistics. Transactions of the Philological Society. 2002;100: 59–129. 10.1111/1467-968X.00091

[5] Gray RD, Greenhill SJ, Atkinson QD. Phylogenetic models of language change: three new questions In: Christiansen MH, Richerson PJ, editors. Cultural Evolution. Cambridge: MIT Press; 2013 pp. 285–300.

[6] Greenhill SJ. Demographic correlates of language diversity In: Bowern C, Evans B, editors. The Routledge Handbook of Historical Linguistics. New York: Routledge; 2015 pp. 557–578.

[7] Lewis MP, Simons GF, Fennig CD. Ethnologue: Languages of the World. 17th edition Dallas: SIL International; 2014 Available: http://www.ethnologue.com

[8] Mayr E. This is biology Cambridge: Harvard University Press; 1998.

[9] Lee K-M, Ramsey SR. A history of the Korean language. Cambridge: Cambridge University Press; 2011.

[10] Sohn H-M. The Korean language. New York: Cambridge University Press; 2001.

[11] Starostin SA, Dybo AV, Mudrak OA. Etymological dictionary of the Altaic languages Leiden: Brill; 2003.

[12] Martin SE. Lexical evidence relating Korean to Japanese. Language. 1966;42: 185–251.

[13] Vovin A. Korea-Japonica Honolulu: University of Hawai'i Press; 2010.

[14] Jin HJ, Tyler-Smith C, Kim W. The peopling of Korea revealed by analyses of mitochondrial DNA and Y-chromosomal markers. PLOS ONE. 2009;4: e4210–10. 10.1371/journal.pone.0004210 19148289 PMC2615218

[15] Kim YJ, Jin HJ. Dissecting the genetic structure of Korean population using genome-wide SNP arrays. Genes Genom. 2013;: 355–363. 10.1007/s13258-013-0082-8

[16] Lee SH, Ffrancon R, Abrams DM, Kim BJ, Porter MA. Matchmaker, matchmaker, make me a match: migration of populations via marriages in the past. Physical Review X. 2014. 10.1103/PhysRevX.4.041009

[17] Lee S, Hasegawa T. Oceanic barriers promote language diversification in the Japanese Islands. J Evol Biol. 2014;27: 1905–1912. 10.1111/jeb.12442 24953224

[18] Axelsen JB, Manrubia S. River density and landscape roughness are universal determinants of linguistic diversity. Proc Biol Sci. 2014;281: 20133029 10.1098/rspb.2013.3029 24741010 PMC4043078

[19] Crowley T, Bowern C. An introduction to historical linguistics Cambridge: Oxford University Press; 2010.

[20] Greenhill SJ, Blust R, Gray RD. The Austronesian Basic Vocabulary Database: from bioinformatics to lexomics. Evol Bioinform. 2008;4: 271–283. 19204825 10.4137/ebo.s893 PMC2614200

[21] Ogura S. Chosengo hogen no kenkyu (A study of Korean dialects) Tokyo: Iwanami Shoten; 1944.

[22] Nanmal ohwi chongbo chori yonguso. Urimal pangen sacen (A dictionary of Korean dialects) Seoul: Nanmal ohwi chongbo chori yonguso; 2010.

[23] Nam K. Kyohak koe sacen (A Middle Korean dictionary) Seoul: Kyohaksa; 2014.

[24] Dunn M. Language phylogenies In: Bowern C, Evans B, editors. The Routledge Handbook of Historical Linguistics. Routledge; 2015 pp. 190–211.

[25] Starostin S, Bronnikov G. Languages of the World Etymological Database: Etymological databases. 2003. Available: http://starling.rinet.ru/cgi-bin/main.cgi?flags=eygtnnl

[26] Ogura S. The outline of the Korean dialects Tokyo: The Toyo Bunko; 1940.

[27] King R. Dialectal variation in Korea In: Sohn H-M, editor. Korean Language in Culture and Society. Honolulu: University of Hawai'i Press; 2006 pp. 264–280.

[28] Drummond AJ, Suchard MA, Xie D, Rambaut A. Bayesian phylogenetics with BEAUti and the BEAST 1.7. Mol Biol and Evol. 2012;29: 1969–1973. 10.1093/molbev/mss075 22367748 PMC3408070

[29] Lee S, Hasegawa T. Bayesian phylogenetic analysis supports an agricultural origin of Japonic languages. Proc Biol Sci. 2011;278: 3662–3669. 10.1098/rspb.2011.0518 21543358 PMC3203502

[30] Lee S, Hasegawa T. Evolution of the Ainu language in space and time. PLOS ONE. 2013;8: e62243–6. 10.1371/journal.pone.0062243 23638014 PMC3637396

[31] Baele G, Lemey P, Bedford T, Rambaut A, Suchard MA, Alekseyenko AV. Improving the accuracy of demographic and molecular clock model comparison while accommodating phylogenetic uncertainty. Mol Biol and Evol. 2012;29: 2157–2167. 10.1093/molbev/mss084 22403239 PMC3424409

[32] Penny D, McComish BJ, Charleston MA, Hendy MD. Mathematical elegance with biochemical realism: the covarion model of molecular evolution. J Mol Evol. 2001;53: 711–723. 10.1007/s002390010258 11677631

[33] Drummond AJ, Ho SYW, Phillips MJ, Rambaut A. Relaxed phylogenetics and dating with confidence. PLOS Biol. 2006;4: e88 10.1371/journal.pbio.0040088 16683862 PMC1395354

[34] Alekseyenko AV, Lee CJ, Suchard MA. Wagner and Dollo: a stochastic duet by composing two parsimonious solos. Syst Biol. 2008;57: 772–784. 10.1080/10635150802434394 18853363 PMC4677801

[35] Huson DH, Bryant D. Application of phylogenetic networks in evolutionary studies. Mol Biol and Evol. 2006;23: 254–267. 10.1093/molbev/msj030 16221896

[36] Pritchard JK, Stephens M, Donnelly P. Inference of population structure using multilocus genotype data. Genetics. 2000;155: 945–959. 10835412 10.1093/genetics/155.2.945 PMC1461096

[37] Hymes D. Pidginization and Creolization of Languages. Camridge: Cambridge University Press; 1974.

[38] Trudgill P. Linguistic and social typology: The Austronesian migrations and phoneme inventories. Linguistic Typology. 2004;8: 305–320. 10.1515/lity.2004.8.3.305

[39] Reesink G, Singer R, Dunn M. Explaining the linguistic diversity of Sahul using population models. PLOS Biol. 2009;7: e1000241 10.1371/journal.pbio.1000241 19918360 PMC2770058

[40] Bowern C. The riddle of Tasmanian languages. Proc Biol Sci. 2012;279: 4590–4595. 10.1098/rspb.2012.1842 23015621 PMC3479735

[41] Evanno G, Regnaut S, Goudet J. Detecting the number of clusters of individuals using the software STRUCTURE: a simulation study. Mol Ecol. 2005;14: 2611–2620. 10.1111/j.1365-294X.2005.02553.x 15969739

[42] Earl DA, vonHoldt BM. STRUCTURE HARVESTER: a website and program for visualizing STRUCTURE output and implementing the Evanno method. Conserv Genet Resour. 2012;4: 359–361. 10.1007/s12686-011-9548-7

[43] Manni F, Guerard E, Heyer E. Geographic patterns of (genetic, morphologic, linguistic) variation: how barriers can be detected by using Monmonier's algorithm. Hum Biol. 2004;76: 173–190. 10.1353/hub.2004.0034 15359530

[44] Brassel KE, Reif D. A procedure to generate Thiessen polygons. Geographical Analysis. 1979;11 10.1111/j.1538-4632.1979.tb00695.x

[45] Voronoï G. Nouvelles applications des paramètres continus à la théorie des formes quadratiques. Deuxième mémoire. Recherches sur les parallélloèdres primitifs. Journal für die reine und angewandte Mathematik. 1908;134: 198–287. 10.1515/crll.1908.134.198

[46] Monmonier MS. Maximum‐difference barriers: An alternative numerical regionalization method. Geographical Analysis. 1973.

[47] R Core Team. R: A language and environment for statistical computing. Vienna, Austria; 2013. Available: http://www.R-project.org

[48] Oksanen J, Blanchet FG, Kindt R, Legendre P, O'hara RB, Simpson GL, et al. vegan: community ecology package. R package version 2.0–7. 2013.

[49] Wickham H. ggplot2: elegant graphics for data analysis New York: Springer; 2009.

[50] Bouckaert R, Heled J. DensiTree 2: seeing trees through the forest. bioRxiv. 2014. 10.1101/012401

[51] Greenhill SJ, Currie TE, Gray RD. Does horizontal transmission invalidate cultural phylogenies? Proc Biol Sci. 2009;276: 2299–2306. 10.1098/rspb.2008.1944 19324763 PMC2677599

[52] Holden CJ, Gray RD. Rapid radiation, borrowing and dialect continua in the Bantu languages In: Forster P, Renfrew C, editors. Phylogenetic Methods and the Prehistory of Languages. Cambridge: McDonald Institute of Archeological Research; 2006 pp. 19–42.

[53] Hong SB, Kim KC, Kim W. Mitochondrial DNA haplogroups and homogeneity in the Korean population. Genes Genom. 2014;36: 583–590. 10.1007/s13258-014-0194-9

[54] Kim SH, Han MS, Kim W, Kim W. Y chromosome homogeneity in the Korean population. Int J Legal Med. 2010;124: 653–657. 10.1007/s00414-010-0501-1 20714743

[55] Hamed MB, Wang F. Stuck in the forest: trees, networks and Chinese dialects. DIA. 2006;23: 29–60. 10.1075/dia.23.1.04ham

[56] Aikhenvald AY, Dixon RMW. Areal diffusion and genetic inheritance. Oxford: Oxford University Press; 2006.

[57] Dixon RMW. The rise and fall of languages New York: Cambridge University Press; 1997.

[58] Anderson MJ, Ellingsen KE, McArdle BH. Multivariate dispersion as a measure of beta diversity. Ecol Lett. 2006;9: 683–693. 10.1111/j.1461-0248.2006.00926.x 16706913

[59] Simpson A. Language and national identity in Asia Oxford: Oxford University Press; 2007.

